# Retinoic acid stimulates meningioma cell adhesion to the extracellular matrix and inhibits invasion

**DOI:** 10.1038/sj.bjc.6690705

**Published:** 1999-10

**Authors:** M Páez Pereda, U Hopfner, U Pagotto, U Renner, E Uhl, E Arzt, C Missale, G K Stalla

**Affiliations:** Department of Endocrinology, Max-Planck Institute of Psychiatry, Kraepelinstr. 10, Munich, 80804, Germany; Department of Neurosurgery, University of Munich, Marchioninistr. 15, Munich, 81377, Germany; Lab. Fisiología y Biología Molecular, Departmento de Biología, FCEN, Ciudad Universitaria Pabellon 2, Buenos Aires, 1428, Argentina; Dept. of Biomedical Sciences and Biotechnology, Div. of Pharmacology, University of Brescia, Via Valsabbina 19, Brescia, 25124, Italy

**Keywords:** meningioma, retinoic acid, tumour invasion, cell adhesion

## Abstract

Meningiomas are tumours derived from the arachnoid and pia mater. During embryogenesis, these membranes develop from the migrating craniofacial neural crest. We have previously demonstrated that meningiomas have characteristic features of embryonic meninges. Craniofacial neural crest derivatives are affected during normal development and migration by retinoic acid. We speculated, therefore, that meningioma cell migration and invasion would be affected in a similar way. In this study we investigated the mechanisms of invasion and migration in meningiomas and the effects of retinoic acid (RA). We found that low doses of RA inhibit in vitro invasion in meningioma cells, without affecting cell proliferation or viability. The matrix metalloproteinases MMP-2 (72 kDa gelatinase) and MMP-9 (92 kDa gelatinase), which play a key role in invasion in other tumours, are not affected by RA. RA inhibits cell migration on collagen I and fibronectin. A possible mechanism for these effects is provided by the fact that RA strongly stimulates adhesion of meningioma cells to extracellular matrix substrates. As in vitro invasion, migration and decreased adhesion to the extracellular matrix correlate with the clinical manifestation of tumour invasion, we conclude that RA induces a non-invasive phenotype in meningioma cells. © 1999 Cancer Research Campaign


British Journal of Cancer (1999) 81(3), 381–386
© 1999 Cancer Research Campaign
Article no. bjoc.1999.0705

### Retinoic acid stimulates meningioma cell adhesion to the extracellular matrix and inhibits invasion

**M Páez Pereda[1], U Hopfner[1], U Pagotto[1], U Renner[1], E Uhl[2], E Arzt[3], C Missale[4] and GK Stalla[1]**

[1]Max-Planck Institute of Psychiatry, Department of Endocrinology, Kraepelinstr. 10, 80804 Munich, Germany; [2]Department of Neurosurgery, University of Munich, Marchioninistr. 15, 81377 Munich, Germany; [3]Lab. Fisiología y Biología Molecular, Departmento de Biología, FCEN, Universidad de Buenos Aires and CONICET, Ciudad Universitaria Pabellon 2, 1428 Buenos Aires, Argentina; [4]Dept. of Biomedical Sciences and Biotechnology, Div. of Pharmacology, University of Brescia, Via Valsabbina 19, 25124 Brescia, Italy

**Summary** Meningiomas are tumours derived from the arachnoid and pia mater. During embryogenesis, these membranes develop from the migrating craniofacial neural crest. We have previously demonstrated that meningiomas have characteristic features of embryonic meninges. Craniofacial neural crest derivatives are affected during normal development and migration by retinoic acid. We speculated, therefore, that meningioma cell migration and invasion would be affected in a similar way. In this study we investigated the mechanisms of invasion and migration in meningiomas and the effects of retinoic acid (RA). We found that low doses of RA inhibit in vitro invasion in meningioma cells, without affecting cell proliferation or viability. The matrix metalloproteinases MMP-2 (72 kDa gelatinase) and MMP-9 (92 kDa gelatinase), which play a key role in invasion in other tumours, are not affected by RA. RA inhibits cell migration on collagen I and fibronectin. A possible mechanism for these effects is provided by the fact that RA strongly stimulates adhesion of meningioma cells to extracellular matrix substrates. As in vitro invasion, migration and decreased adhesion to the extracellular matrix correlate with the clinical manifestation of tumour invasion, we conclude that RA induces a non-invasive phenotype in meningioma cells. © 1999 Cancer Research Campaign

**Keywords:** meningioma; retinoic acid; tumour invasion; cell adhesion

Meningiomas are usually benign intracranial and intraspinal tumours. Invasive meningiomas can, however, penetrate the brain parenchyma and disturb vital structures. The incidence of hemiparesis or neurological defects is higher in patients with invasive meningiomas (Akeyson and McCutcheon, 1996). Post-operative survival is 9 years on average for benign meningiomas, whereas it can be as low as 7 months for invasive meningiomas (Akeyson and McCutcheon, 1996). Tumour invasion of the meninges and the brain requires the digestion of the surrounding extracellular matrix (ECM). Collagen and fibronectin are most abundant in normal dura mater at the limits between neuroepithelial and meningeal elements, and also among meningioma cells (Rutka et al, 1987; Nitta et al, 1990).

Meningioma cells derive from the arachnoid and pia mater (Black, 1993). These membranes derive, in turn, from the craniofacial neural crest, which migrates around the anterior neural tube and colonizes the head mesenchyma (Murphy and Bartlett, 1993). We have previously shown that meningiomas express endothelins and endothelin receptors (Pagotto et al, 1995). This autocrine loop controls meningioma cell proliferation and may play an important role in meningioma pathogenesis. In analogy with this, the expression of this endothelin autocrine loop was also shown to be important during the embryonic development of the normal meninges (Kurihara et al, 1994). Therefore, we speculate that meningiomas express similar genetic programmes as the fetal meninges. Besides the control of proliferation, one particularly intriguing possibility is that the migration and invasion in tumours derived from the meninges might involve the same mechanisms that occur during the migration of the craniofacial neural crest cells in normal development.

Craniofacial neural crest has been shown to be highly sensitive to retinoic acid (RA) (Marshall et al, 1996; Morris-Kay and Sokolova, 1996). RA inhibits the migration of the anterior neural crest (Lee et al, 1995; Gale et al, 1996). Some of the effects of RA on neural crest cell migration are related to changes in the ECM composition (Moro Balbas et al, 1993). RA also changes and transforms the identity of neural crest cells by regulating the expression of regulatory genes such as the homeobox transcription factors (Marshall et al, 1992). It is assumed that these transcription factors would in turn regulate the expression of target effector genes, including the migration machinery.

RA has also been shown to inhibit the growth of many types of cancer in different models (Lotan, 1996). Although RA blocks cell transformation, induces cell differentiation and controls apoptosis and proliferation in vitro in other cell types (Harisiadis et al, 1978; Bertram, 1983), the mechanisms of RA inhibition of tumour development in vivo are still not fully understood. RA seems to act in vivo in the late stages of tumour progression rather than in transformation and proliferation (McGarvey et al, 1990). In agreement with this, it has been shown that RA inhibits melanoma cell migration, adhesion and in vitro invasion (Helige et al, 1993; Situ et al, 1993; Jakob et al, 1998). RA chemopreventive effects can also be related to its influence on cell–cell and cell–substrate interactions (Hossein et al, 1989; Hossein and Bertram, 1994; Toyofuku et al, 1998).

In the present study we examined the cellular mechanisms of invasion in meningioma cells in correlation with the clinical manifestation of invasion, and describe the effects of RA on meningioma invasion, migration and adhesion.

*Received 27 October 1998*
*Revised 9 March 1999*
*Accepted 11 March 1999*

*Correspondence to*: M Páez Pereda

**Table 1** Retinoic acid inhibits meningioma cell invasion

| | In vitro invasion (invasive cells per field) | |
|---|---|---|
| | Control | $10^{-8}$ mol $l^{-1}$ retinoic acid*[a] |
| 1 | 52 ± 6 | 22 ± 3 |
| 2 | 25 ± 2 | 18 ± 1 |
| 3 | 19 ± 3 | 10 ± 1 |
| 4 | 37 ± 2 | 30 ± 2 |
| 5 | 22 ± 3 | 7 ± 1 |
| 6 | 28 ± 4 | 15 ± 2 |
| 7 | 138 ± 16 | 58 ± 4 |
| 8 | 98 ± 7 | 23 ± 4 |
| 9 | 79 ± 9 | 55 ± 7 |
| 10 | 85 ± 6 | 53 ± 4 |

[a]In all cases the differences between treatments are statistically significant ($P < 0.01$, ANOVA combined with Scheffé's test). The last four cases correspond to invasive meningiomas.

**Table 2** Retinoic acid does not affect TIMP-1 production

| | TIMP-1 (ng $ml^{-1}$) | |
|---|---|---|
| | Control | $10^{-8}$ mol $l^{-1}$ retinoic acid[a] |
| 1 | 27.3 ± 1.2 | 22.5 ± 3.3 |
| 2 | 55.3 ± 2.9 | 58.2 ± 1.5 |
| 3 | 43.7 ± 3.2 | 40 ± 5.3 |
| 4 | 20 ± 2.8 | 20.6 ± 2.3 |
| 5 | 32.9 ± 3.5 | 35 ± 4.2 |
| 6 | 82 ± 6.9 | 85 ± 5.7 |
| 7 | 87 ± 5.7 | 88 ± 4.3 |
| 8 | 28.6 ± 2.3 | 25 ± 3.5 |
| 9 | 69 ± 5.6 | 71 ± 7.6 |

[a] In all cases the differences between treatments are not significant ($P <$ 0.01, ANOVA combined with Scheffé's test). The last three cases correspond to invasive meningiomas.

### MATERIALS AND METHODS

### Materials

Unless stated, all reagents were from Sigma (St Louis, MO, USA), Boehringer (Mannheim, Germany) or Pharmacia (Uppsala, Sweden).

### Meningioma cell culture

The cell culture reagents and materials were purchased from Gibco-BRL Life Technologies (Eggenstein, Germany), Seromed (Berlin, Germany), or Nunc (Wiesbaden, Germany). Tumour samples were obtained from 30 patients with intracranial meningiomas. Invasion was assessed by nuclear magnetic resonance, direct intraoperative observation of the tumour and surrounding tissue, and histological evaluation. Other cases in which the diagnosis of invasiveness was inconclusive were excluded from this study. The 19 non-invasive meningiomas were classified histologically according to the World Health Organization (Zülch, 1979) as meningotheliomatous (13), fibroblastic (two), transitional (three) and anaplastic (one). The 11 invasive meningiomas were classified as meningotheliomatous (four), fibroblastic (three), transitional (two), psammomatous (one) and anaplastic (one).

Meningioma cell culture was performed as previously described (Pagotto et al, 1995). Briefly, meningioma tissue was rinsed three times in preparation buffer (137 mmol $l^{-1}$ sodium chloride, 5 mmol $l^{-1}$ potassium chloride, 0.7 mmol $l^{-1}$ $Na_2HPO_4$, 15 mmol $l^{-1}$ HEPES pH 7.3, 10 mmol $l^{-1}$ glucose, 2.5 mg $l^{-1}$ amphotericin-B, and $10^5$ U $l^{-1}$ penicilin–streptomycin) and then dissected into small pieces. The tissue fragments were incubated in preparation buffer with the addition of 1000 U $ml^{-1}$ collagenase (Worthington Biochemical Corporation, Freehold, NJ, USA), 2 g $l^{-1}$ hyaluronidase 4 g $l^{-1}$ bovine serum albumin (BSA), 10 mg $l^{-1}$ DNAase II and 1 g $l^{-1}$ soybean trypsin inhibitor with gentle rocking at 37°C until dispersed. The cells were resuspended in culture medium: Dulbecco's modified Eagle's medium (DMEM) pH 7.3 containing 10% fetal calf serum, 2.2 g $l^{-1}$ sodium hydrogen carbonate ($NaHCO_3$), 10 mmol $l^{-1}$ HEPES, 2 mmol $l^{-1}$ glutamine, 10 ml $l^{-1}$ non-essential amino acids, 10 ml $l^{-1}$ modified essential medium (MEM) vitamins, 5 mg $l^{-1}$ insulin, 5 mg $l^{-1}$ transferrin, 2.5 mg $l^{-1}$ amphotericin-B, $10^5$ U $l^{-1}$ penicilin–streptomycin, 20 mg $ml^{-1}$ sodium selenit, and 30 pmol $l^{-1}$ $T_3$ (Henning, Germany). The cell viability was routinely over 90% as assessed by acridine orange–ethidium bromide staining. The cells were incubated for 1 week under 5% carbon dioxide atmosphere at 37°C and used at the first passage. The differentiation state of the cultures was routinely checked as previously described (Pagotto et al, 1995). The culture medium was replaced by stimulation medium (DMEM pH 7.3 containing 2.2 g $l^{-1}$ $NaHCO_3$, 10 mmol $l^{-1}$ HEPES, 2 mmol $l^{-1}$ glutamine and 1 g $l^{-1}$ BSA) for 24 hours. Then, $10^{-8}$ mol $l^{-1}$ *all-trans*-RA (Sigma, St Louis, MO, USA) was added. After 24 h, the medium was collected for zymographic analysis and total protein measurements. The cells were collected with citrate buffer and then used for in vitro invasion, migration and invasion assays. The cell viability after RA treatment was checked by acridine orange–ethidium bromide staining. Only concentrations higher than $10^{-6}$ mol $l^{-1}$ RA were found to be toxic for these cells.

### In vitro invasion

The invasive potential of meningioma cells was analysed as we previously described for small-cell lung cancer cells (Missale et al, 1998). Polycarbonate filters, with 8-µm pore size, in 24-transwell chambers were covered with 30 µg of Matrigel (Becton Dickinson, Heidelberg, Germany) and dried. Then 100 000 cells were added in DMEM containing 0.1% BSA to the inner section of the transwell. Fetal calf serum (0.5%) was used as chemoattractant in the outer chamber. After a 24-h incubation, the cells in the inner chamber were removed with a cotton swab. The cells attached to the bottom side of the membrane were fixed with 4% formaldehyde, stained with haematoxylin and counted. The cell counting was always performed by the same operator who had no previous knowledge of the clinical diagnosis.

### Cell migration

The in vitro migration of meningioma cells was analysed as previously described (Ledda et al, 1997). Twenty thousand cells were seeded in Boyden chambers on polycarbonate filters covered with 5 µg $cm^{-2}$ collagen I or fibronectin. Fetal calf serum (0.5%) was used as a chemoattractant in the lower chamber. After a 24-h incubation, the cells in the inner chamber were removed with a cotton swab. The cells attached to the bottom side of the membrane were fixed with 4% formaldehyde, stained with haematoxylin and counted.

 © 1999 Cancer Research Campaign

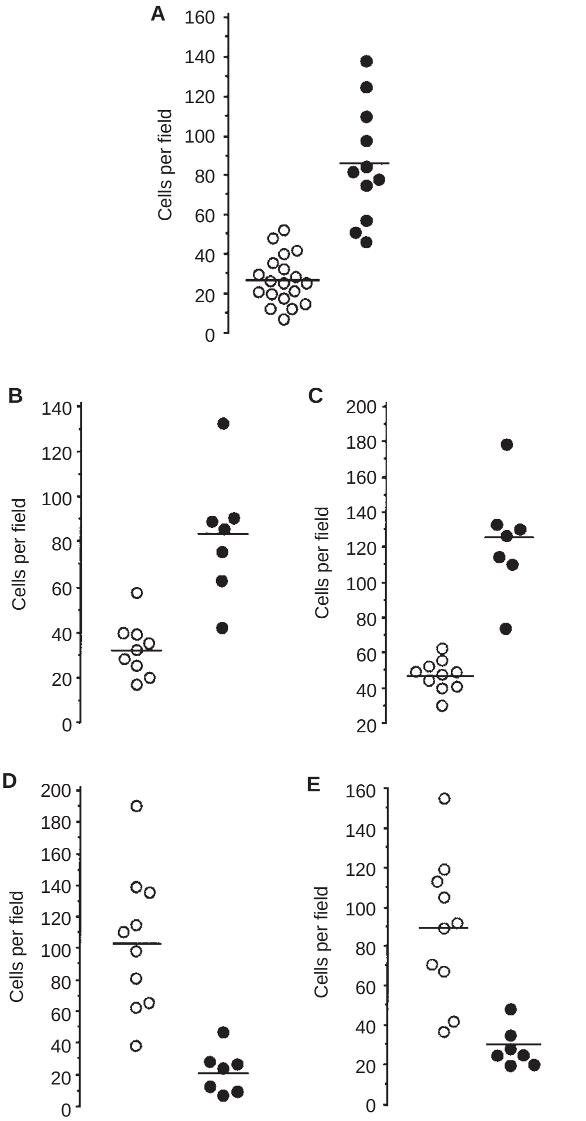

**Figure 1** In vitro parameters of meningioma cell invasion, migration and adhesion to the extracellular matrix correlate with clinical parameters of invasion. (**A**) In vitro invasion was measured in a series of meningiomas as detailed in Materials and Methods. (**B**) Meningioma cell migration on a collagen I substrate. (**C**) Meningioma cell migration on a fibronectin substrate. (**D**) Short-term cell adhesion to a collagen I substrate. (**E**) Short-term cell adhesion to a fibronectin substrate. Full circles: clinically invasive meningiomas, open circles: non-invasive meningiomas. Horizontal lines indicate the mean

### Short-term cell adhesion

Ten thousand cells per well were seeded into 96-well plates previously coated with collagen I or fibronectin (Becton Dickinson, Heidelberg, Germany). After a 3-h incubation, the plates were shaken for 1 min at 150 rpm and washed with phosphate-buffered saline (PBS) to remove any non-adherent cells. The attached cells were fixed with 4% formaldehyde, stained with haematoxylin and counted.

### Zymographic analysis of gelatinases

Gelatinolytic activity was analysed as described (Ledda et al, 1997) using 10% polyacrylamide gels containing 0.2% gelatin. Conditioned media obtained as described earlier were mixed with loading buffer containing 2.5% sodium dodecyl sulphate (SDS) and incubated for 30 min at room temperature before loading onto the gel. After electrophoresis at 4°C under non-denaturing conditions, the gels were washed as described (Ledda et al, 1997). After staining with Coomassie blue R250, gelatinase activity was observed as clear zones of proteolysis against a blue background. Incubation of the gels in the presence of 20 mmol $l^{-1}$ EDTA was performed to demonstrate the calcium ion and zinc ion dependence of the proteinase activity observed. Human fetal fibroblast (HFL-1) cells and melanoma cells (MDA-MB231) were used as positive controls (Ledda et al, 1997).

### Statistics

Statistics were performed using one-way analysis of variance (ANOVA) in combination with the Scheffé's test. The results are expressed as mean ± s.e.m.

### RESULTS

### Invasion, migration and adhesion in meningioma cells

The invasive potential of meningioma cells was measured in 30 different cell preparations. Different tumours showed different in vitro invasive potentials. The invasive ability of meningioma cells correlated with the clinical and histological parameters of invasion (Figure 1A). The invasive tumours showed the highest in vitro invasive potential, whereas the less aggressive ones showed significantly lower invasion rates (Figure 1A). We measured cell migration on ECM-coated membranes and found that the more aggressive meningiomas had higher migration rates on collagen I and fibronectin (Figure 1 B, C). To study whether these differences in invasion and migration are related to the adhesion to ECM components we measured short-term cell adhesion to collagen I and fibronectin-coated plates (Palacek et al, 1997). We found that the invasive meningiomas show significantly lower adhesion to ECM components than the benign ones (Figure 1 D, E). Therefore, there is a positive correlation between the clinical manifestation of invasiveness, the in vitro invasive potential and the in vitro cell migration. Accordingly, there is a negative correlation between the clinical observations and the cell adhesion to collagen I and fibronectin substrates.

### Gelatinase activity in meningiomas

Invasion requires the digestion of extracellular matrix components by matrix metalloproteinases (MMP). Among these, MMP-2 and MMP-9 are expressed early during the acquisition of the invasive phenotype and correlate with increased invasion and metastasis (Stetler-Stevenson et al, 1993; Stetler-Stevenson, 1996). We analysed the activity of MMP-2 and MMP-9 by gelatine zymogram in 20 meningioma cell cultures (13 non-invasive and seven invasive). The different tumours displayed different gelatinase activity patterns (Figure 2). The different bands were identified as proMMP-9, MMP-9, proMMP-2, and MMP-2. The 66-kDa band co-migrated with the proMMP-2 secreted by HFL-1 cells. The second band was confirmed as the active form of MMP-2 because it co-migrated with a positive control obtained from HFL-1-conditioned medium incubated with MDA-MB231 cells treated with Concanavalin A, which was shown to activate MMP-2. The identity of the bands as metalloproteinases

*© 1999 Cancer Research Campaign*

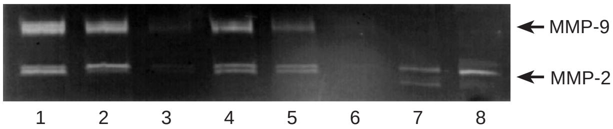

**Figure 2** Matrix metalloproteinase activity in different meningiomas. Meningioma cells were cultured and the medium collected after 24 h for zymogram analysis as described in Materials and Methods. Lanes 1, 3 and 6, meningotheliomatous; lanes 2 and 7, fibroblastic; lanes 4 and 8, transitional; lane 5 anaplastic meningiomas. Lanes 1, 4, 5 and 8, invasive; lanes 2, 3, 6 and 7, non-invasive meningiomas.

**Table 3** Retinoic acid inhibits cell migration on collagen I and fibronectin

| | Cell migration (migrating cells/field) | | | |
|---|---|---|---|---|
| | Collagen I | | Fibronectin | |
| | Control | $10^{-8}$ mol $l^{-1}$ retinoic acid[a] | Control | $10^{-8}$ mol $l^{-1}$ retinoic acid[a] |
| 1 | 57 ± 7 | 29 ± 3 | 62 ± 5 | 22 ± 2 |
| 2 | 25 ± 3 | 14 ± 3 | 40 ± 6 | 17 ± 3 |
| 3 | 35 ± 3 | 19 ± 2 | 45 ± 5 | 17 ± 2 |
| 4 | 28 ± 4 | 12 ± 2 | 52 ± 5 | 16 ± 2 |
| 5 | 40 ± 5 | 27 ± 1 | 50 ± 7 | 29 ± 4 |
| 6 | 39 ± 3 | 18 ± 2 | 50 ± 3 | 22 ± 1 |
| 7 | 121 ± 15 | 34 ± 5 | 180 ± 21 | 90 ± 11 |
| 8 | 75 ± 9 | 48 ± 4 | 132 ± 25 | 43 ± 3 |
| 9 | 85 ± 4 | 42 ± 4 | 117 ± 19 | 49 ± 6 |
| 10 | 90 ± 11 | 36 ± 5 | 135 ± 30 | 38 ± 5 |

[a]In all cases the differences between treatments are statistically significant ($P < 0.01$, ANOVA combined with Scheffé's test). The last four cases correspond to invasive meningiomas.

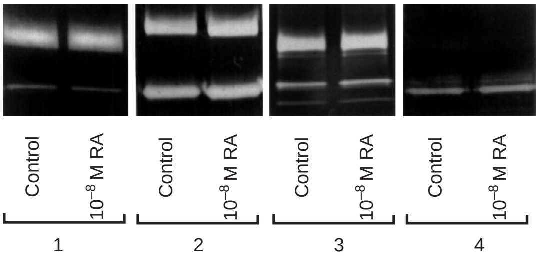

**Figure 3** Retinoic acid does not alter matrix metalloproteinase activity in meningiomas. Meningioma cells were cultured and treated with $10^{-8}$ mol $l^{-1}$ retinoic acid for 24 h. Conditioned media were collected at the end of the treatment for zymogram analysis as described in Materials and Methods. Lanes 1 and 2, meningotheliomatous; lane 3, fibroblastic; lane 4, psamomatous meningiomas. Lanes 1, 2 and 3, non-invasive; lane 4, invasive meningioma was further confirmed by inhibition with EDTA (data not shown). The intensity of the bands or the presence of the active forms of the enzymes did not seem to correlate with the tumour invasion. Therefore, in meningiomas, gelatinase activity seems not to be a limiting step for the clinical manifestation of invasiveness.

### RA inhibits meningioma invasion

RA treatment in low doses ($10^{-8}$ mol $l^{-1}$) significantly inhibited in vitro invasion of meningioma cells as measured by Boyden chamber assays (Table 1). All the cases analysed ($n$ = 10) showed

**Table 4** Retinoic acid does not produce rapid changes in cell adhesion

| | Cell adhesion (attached cells per field) | | | |
|---|---|---|---|---|
| | Collagen I | | Fibronectin | |
| | Control | $10^{-8}$ mol $l^{-1}$ retinoic acid | Control | $10^{-8}$ mol $l^{-1}$ retinoic acid |
| 2 | 24 ± 1 | 23 ± 3 | 22 ± 2 | 25 ± 3 |
| 3 | 19 ± 2 | 17 ± 1 | 15 ± 1 | 13 ± 3 |
| 4 | 12 ± 1 | 12 ± 2 | 14 ± 1 | 13 ± 2 |
| 8 | 3 ± 1 | 3 ± 2 | 3 ± 2 | 4 ± 2 |

**Table 5** Retinoic acid stimulates cell adhesion to collagen I and fibronectin

| | Cell adhesion (attached cells per field) | | | |
|---|---|---|---|---|
| | Collagen I | | Fibronectin | |
| | Control | $10^{-8}$ mol $l^{-1}$ retinoic acid[a] | Control | $10^{-8}$ mol $l^{-1}$ retinoic acid[a] |
| 1 | 14 ± 2 | 47 ± 3 | 21 ± 2 | 77 ± 5 |
| 2 | 14 ± 1 | 77 ± 8 | 12 ± 2 | 35 ± 3 |
| 3 | 19 ± 2 | 93 ± 10 | 15 ± 1 | 93 ± 10 |
| 4 | 11 ± 1 | 55 ± 7 | 10 ± 1 | 53 ± 7 |
| 5 | 9 ± 2 | 65 ± 5 | 9 ± 2 | 39 ± 4 |
| 6 | 12 ± 3 | 49 ± 6 | 7 ± 3 | 60 ± 7 |
| 7 | 3 ± 1 | 14 ± 2 | 4 ± 2 | 13 ± 2 |
| 8 | 3 ± 1 | 19 ± 3 | 5 ± 2 | 21 ± 3 |
| 9 | 5 ± 1 | 28 ± 4 | 3 ± 1 | 18 ± 1 |
| 10 | 3 ± 2 | 9 ± 2 | 3 ± 2 | 15 ± 2 |

[a]In all cases the differences between treatments are statistically significant ($P < 0.01$, ANOVA combined with Scheffé's test). The last four cases correspond to invasive meningiomas.

this inhibitory response irrespective of the basal level of invasion or the clinical diagnosis. In the same conditions, retinoic acid did not produce any changes in cell viability or proliferation measured by thymidine incorporation, 3-(4,5-dimethylthiazol-2-yl)-2,5-diphenyltetrazolium bromide (MTT) or vital staining (data not shown). This indicates that the inhibitory effects of retinoic acid are not due to a cytotoxic effect. In parallel, MMP-2 and MMP-9 activity was measured in 12 meningiomas of different types treated with RA (Figure 3). RA did not affect MMP-2 or MMP-9 activity in the same tumours which showed responses in in vitro invasion. Since metalloproteinase activity also depends on the secretion of tissue inhibitors of metalloproteinases (TIMPs) (Stetler-Stevenson, 1996), we analysed by enzyme-linked immunosorbent assay the effects of RA on TIMP-1 expression. RA did not produce any effect on TIMP-1 expression in nine cases analysed (Table 2). Therefore, MMP-2 and MMP-9 activity is probably not involved in the effects of RA on invasion.

### RA inhibits meningioma cell migration

Besides digestion of ECM components, another step of the invasion process is cell migration. We studied the migration of meningioma cells on ECM substrates and the effects of RA. RA inhibited the migration of meningioma cells on Boyden chambers covered with collagen I and fibronectin, major components of the interstitial ECM (Table 3). RA does not affect the migration on

 © 1999 Cancer Research Campaign polycarbonate substrate (data not shown). This rules out the possibility that RA affects the response of the cells to chemoattractants.

### RA stimulates adhesion to the ECM

Cell migration depends on cell adhesion to the ECM. As an integrative and quantitative parameter, we measured short-term cell adhesion to ECM substrates (Palacek et al, 1997). To rule out a direct effect of RA on cell adhesion at the level of the cell membrane we added $10^{-8}$ mol $l^{-1}$ RA just before the adhesion assay. No significant differences were observed in cell adhesion under these conditions (Table 4). This indicates that RA does not affect cell adhesion through a direct effect on the cell membrane. However, after a 24-h treatment, RA strongly increased short-term cell adhesion to collagen I and fibronectin in all the cases analysed ($n$ = 10) (Table 5). This enhanced adhesion to the ECM substrates is in agreement with the inhibition of cell motility observed in the migration and invasion assays.

### DISCUSSION

In this study we examined the cellular mechanisms of invasion in human meningiomas and investigated the effects of RA on in vitro invasion, migration and adhesion. We report that the clinical manifestation of tumour invasion correlates with in vitro invasion, cell migration and a decreased adhesion of meningioma cells to ECM substrates. In addition, we demonstrate for the first time that RA inhibits in vitro invasion and migration of meningioma cells and these effects involve an increase in the adhesion to ECM components. Therefore, we speculate that RA could revert the invasive meningiomas to a non-invasive phenotype similar to the one of clinically non-invasive tumours.

RA has been shown to successfully inhibit tumour development in several models (Lotan, 1996). It inhibits transformation and proliferation, induces differentiation and apoptosis in vitro, and inhibits tumour growth in vivo (Harisiadis et al, 1978; Bertram et al, 1983; McGarvey et al, 1990). Here we show that RA also affects later stages of tumour development such as migration and invasion. These effects could possibly involve an increased adhesion to the ECM components. Our results therefore provide further insight into the cellular mechanisms involved in the effects of RA on tumour development.

Tumour invasion is a complex multistep process that requires proteolysis of the ECM performed by MMPs, dynamic changes in cell adhesion and cell migration. MMP-2, MMP-9 and their inhibitor TIMP-1 which play an important role in the invasion of many aggressive types of cancer (Stetler-Stevenson et al, 1993) do not seem to be involved in meningioma invasion. RA has been shown to inhibit MMP expression in different cell types (Woessner, 1991). However, in meningioma cells, RA did not produce any changes in MMP-2, MMP-9 or TIMP-1. Similar effects of RA on invasion without affecting MMP-2 and TIMP-1 expression were recently observed in melanoma cells (Jacob et al, 1998). Although we cannot exclude the participation of other metalloproteinases, the changes in in vitro invasion produced by RA in meningioma cells are probably not due to a reduced degradation of the ECM. Moreover, the strong stimulation of cell adhesion and the reduced migration on ECM substrates indicate that RA mainly affects cell motility in meningiomas.

It is well established that RA causes craniofacial malformations when administered during development (Durston et al, 1989; Gale et al, 1996; Marshall et al, 1996; Morris-Kay and Sokolova, 1996). These effects are produced by changes in the identity and migration patterns of the neural crest cells (Marshall et al, 1992). It was previously shown that RA alters the proteoglycan composition of the ECM thereby affecting neural crest cell migration during development (Moro Balbas et al, 1993). These results are in agreement with our observations that retinoic acid modifies the cell–substrate interactions in the neural crest-derived meningioma cells, probably by regulating the levels of integrin expression or the affinity for the ECM components.

We have previously shown that meningioma cells, in contrast to normal adult meninges, express an endothelin autocrine loop (Pagotto et al, 1995). Endothelin signalling is necessary for the post-migratory proliferation of the neural crest cells but does not affect cell migration (Clouthier et al, 1998). This embryonic regulatory system controls the normal development of the meninges and could play a role in the pathological transformation of meningiomas (Kurihara et al, 1994). The similarity between meningioma cells and fetal meninges, together with the present results of the effects of RA on invasion and migration, support the concept that meningioma cells express a fetal genetic programme that could be involved in the mechanisms governing meningioma pathogenesis at the level of proliferation and invasion.

As in vitro invasion, migration and decreased adhesion to the ECM correlate with the clinical manifestation of invasiveness we conclude that retinoic acid induces a non-invasive phenotype in meningioma cells. This suggests that RA therapy may benefit patients with meningiomas, where complete surgical resection is not possible, and thus prevent the invasion of the normal surrounding tissue.

### ACKNOWLEDGEMENTS

We wish to thank Dr P Largen for reviewing the manuscript for English usage. This work was supported by a grant from the Deutsche Forschungsgemeinschaft (Sta 285/10-1, 10-2).

### REFERENCES

Akeyson EW and McCutcheon IE (1996) Management of benign and aggressive intracranial meningiomas. *Oncology* **10**: 747–759

Bertram JS (1983) Inhibition of neoplastic transformation in vitro by retinoids. *Cancer Surv* **3**: 243–262

Black PM (1993) Meningiomas. *Neurosurgery* **32**: 643–657

Clouthier DE, Hosoda K, Richardson JA, Williams SC, Yanagisawa H, Kuwaki T, Kumada M, Hammer RE and Yanagisawa (1998) Cranial and cardiac neural crest defects in endothelin-A receptor deficient mice. *Development* **125**: 813–824

Durston AJ, Timmermans JPM, Hage WJ, Hendriks HFJ, DeVries NJ, Heideveld M and Niewkoop PD (1989) Retinoic acid causes an anteroposterior transformation in the developing central nervous system. *Nature* **340**: 140–144

Gale E, Prince V, Lumsde A, Clarke J, Holder N and Maden M (1996) Late effects of retinoic acid on neural crest and aspects of rhombomere. *Development* **122**: 783–793

Harisiadis L, Miller RC, Hall EJ and Borek C (1978) A vitamin A analogue inhibits radiation-induced oncogene transformation. *Nature* **174**: 186–187

Helige C, Smolle J, Zellnig G, Hartmann E, Fink-Puches R, Kerl H and Tritthart HA (1993) Inhibition of K1735-M2 melanoma cell invasion in vitro by retinoic acid. *Clin Exp Metastasis* **11**: 409–418

Hossein MZ and Bertram JS (1994) Retinoids suppress proliferation, induce cell spreading, and up-regulate connexin 43 expression only in postconfluent 10T1/2 cells: implications for the role of gap junctional communication. *Cell Growth Differ* **5**: 1253–1261

Hossein MZ, Wilkens LR, Mehta PP, Loewenstein W and Bertram JS (1989) Enhancement of gap junctional communication by retinoids correlates with

© 1999 Cancer Research Campaign their ability to inhibit neoplastic transformation. *Carcinogenesis* **10**: 1743–1748

Jacob K, Wach F, Holzapfel U, Hein R, Lengyel E, Buettner R and Bosserhoff AK (1998) In vitro modulation of human melanoma cell invasion and proliferation by all-trans-retinoic acid. *Melanoma Res* **8**: 211–219

Kurihara Y, Kurihara H, Suzuki H, Kodama T, Maemura K, Nagai R, Oda H, Kuwaki T, Cao W, Kamada N, Jishage K, Ouchi Y, Azuma S, Toyoda Y, Ishikawa T, Kumada M and Yazaki Y (1994) Elevated blood pressure and craniofacial abnormalities in mice deficient in endothelin-1. *Nature* **368**: 703–710

Ledda MF, Adris S, Bravo Al, Kairiyama C, Bover L, Chernajovsky Y, Mordoh S and Podhajcer OL (1997) Suppression of SPARC expression by antisense RNA abrogates the tumorigenicity of human melanoma cells. *Nat Med* **3**: 171–176

Lee YM, Osumi-Yamashita N, Ninomiya Y, Moon CK, Eriksson U and Eto K (1995) Retinoic acid stage-dependently alters the migration pattern and identity of hindbrain neural crest cells. *Development* **121**: 825–837

Lotan R (1996) Retinoids in cancer chemoprevention. *FASEB J* **10**: 1031–1039

McGarvey TW, Silberman S and Persky B (1990) The effect of butyric acid and retinoic acid on invasion and experimental metastasis of murine melanoma cells. *Clin Exp Metastasis* **8**: 433–448

Mahmood A, Caccamo DV, Tomecek FJ and Malik GM (1993) Atypical and malignant meningiomas: a clinicopathological review. *Neurosurgery* **33**: 955–963

Marshall H, Nonchev S, Sham MH, Muchamore I, Lumsden A and Krumlauf R (1992) Retinoic acid alters hindbrain Hox code and induces transformation of rhombomeres 2/3 into a 4/5 identity. *Nature* **360**: 737–741

Marshall H, Morrison A, Studer M, Pöpperl H and Krumlauf R (1996) Retinoids and Hox genes. *FASEB J* **10**: 969–978

Missale C, Codignola A, Sigala S, Finardi A, Paez-Pereda M, Sher E and Spano PF (1998) Nerve growth factor abrogates the tumorigenicity of human small cell lung cancer cell lines. *Proc Natl Acad Sci USA* **95**: 5366–5371

Moro Balbas JA, Gato A, Alonso Revuelta MI, Pastor JF, Repressa JJ and Barbosa E (1993) Retinoic acid induces changes in the rhomboencephalic neural crest cells migration and extracellular matrix composition in chick embryos. *Teratology* **48**: 197–206

Morris-Kay GM and Sokolova N (1996) Embryonic development and pattern formation. *FASEB J* **10**: 961–968

Murphy M and Bartlett PF (1993) Molecular regulation of neural crest development. *Mol Neurobiol* **7**: 111–135

Nitta H, Yamashima T, Yamashita J and Kubota T (1990) An ultrastructural and immunohistochemical study of extracellular matrix in meningiomas. *Histol Histopathol* **5**: 267–274

Pagotto U, Arzberger T, Hopfner U, Sauer J, Renner U, Newton CJ, Lange M, Uhl E, Weindl A and Stalla GK (1995) Expression and localisation of endothelin-1 and endothelin receptors in human meningiomas. Evidence for a role in tumoral growth. *J Clin Invest* **96**: 2017–2025

Palacek SP, Loftus JC, Ginsberg MH, Lauffenburger DA and Horwitz AF (1997) Integrin-ligand binding properties govern cell migration speed through cell–substratum adhesiveness. *Nature* **385**: 537–540

Rutka JT, Myatt CA, Giblin JR, Davis RL and Rosenblum ML (1987) Distribution of extracellular matrix proteins in primary human brain tumors: an immunohistochemical analysis. *Can J Neurol Sci* **14**: 25–30

Situ R, Inman DR, Fligiel SE and Varani J (1993) Effects of all-trans-retinoic acid on melanocyte adhesion and motility. *Dermatology* **186**: 38–44

Stetler-Stevenson WG (1996) Dynamics of matrix turnover during pathological remodelling of the extracellular matrix. *Am J Pathol* **148**: 1345–1350

Stetler-Stevenson WG, Aznavoorian S and Liotta L (1993) Tumor cell interactions with the extracellular matrix during invasion and metastasis. *Annu Rev Cell Biol* **9**: 541–573

Toyofuku T, Yabuki M, Otsu K, Kuzuya T, Hori M and Tada M (1998) Direct association of the gap junction protein connexin-43 with ZO-1 in cardiac myocytes. *J Biol Chem* **273**: 12725–12731

Woessner JF Jr (1991) Matrix metalloproteinases and their inhibitors in connective tissue remodelling. *FASEB J* **5**: 2145–2154

Zülch KG (1979) Histological typing of tumors of the central nervous system. In: *International Histologic Classification of Tumors*, No. 21, pp 47–60. World Health Organization: Geneva

*© 1999 Cancer Research Campaign*
